# Precision intervention based on infection site: strategies and advances of magnetic nanomaterials in bacterial therapy

**DOI:** 10.3389/fchem.2026.1794622

**Published:** 2026-04-22

**Authors:** Guangxin Zhang, Peiyi Liang, Xiying Fu, Yicun Wang

**Affiliations:** 1 Department of Thoracic Surgery, Second Hospital of Jilin University, Changchun, China; 2 Department of Medical Research Center, Second Hospital of Jilin University, Changchun, China; 3 Department of Endocrinology, Second Hospital of Jilin University, Changchun, China

**Keywords:** bacterial infection, biofilm disruption, magnetic nanoparticles, magnetothermal effect, photothermal effect, stimuli-responsive nanomaterials, targeting

## Abstract

Bacterial infections, especially those involving drug-resistant pathogens and biofilms, pose a severe global health threat. Conventional antibiotic therapies are limited by poor penetration, low specificity, and bacterial resistance mechanisms. Magnetic nanoparticles (MNPs) offer a promising alternative by combining magnetically guided targeting, magnetothermal/photothermal effects, multifunctional drug delivery, and imaging capabilities. Their antibacterial efficacy depends critically on the anatomical and pathological features of the infection site. For skin and superficial infections, near-infrared (NIR) light, particularly in the second biological window (NIR-II), enables synergistic photothermal/photodynamic/chemodynamic therapies. For deep soft tissue and bone infections, alternating magnetic fields (AMF) provide deep-penetrating magnetothermal activation or targeted enrichment, often combined with image-guided intervention. For cavity organ and implant-related infections, surface functionalization, local drug delivery, and endoscopic energy application allow precise interfacial intervention. This review systematically discusses MNP-based strategies tailored to different infection sites, integrating advances in material design, synergistic mechanisms, and preclinical progress. It also addresses challenges in multifunctional integration, biosafety, and clinical translation, and outlines future directions toward intelligent, theranostic, and synergistic antibacterial platforms.

## 1 Introduction

Bacterial infections remain a major global health challenge, primarily due to antimicrobial resistance (AMR) and biofilm formation. According to the Global Burden of Disease Study 2019, infectious diseases accounted for approximately 13.7 million deaths worldwide, while bacterial infections were directly responsible for an estimated 7.7 million deaths annually (GBD, 2019 Antimicrobial Resistance Collaborators, 2022). These infections contributed to millions disability-adjusted life years (DALYs), highlighting their profound impact on global health systems and socioeconomic development. Lower respiratory infections, tuberculosis, enteric infections, and sepsis remain among the leading contributors to infection-related DALYs, particularly in low- and middle-income countries.

Biofilms not only enhance bacterial tolerance to drugs but also impede immune clearance, making infections chronic and difficult to eradicate (Ghanayem et al., 2025; Ortega-Portas and Esteban, 2025). Pathogens such as *Pseudomonas aeruginosa*, carbapenem-resistant *Acinetobacter* baumannii, and methicillin-resistant *Staphylococcus aureus* (*MRSA*) further limit the efficacy of conventional antibiotics (Goenka et al., 2025; Quan et al., 2020). With the declining pipeline of new antibiotics, innovative strategies are urgently needed to overcome biofilm barriers and resistance mechanisms.

Nanotechnology, particularly magnetic nanoparticles (MNPs), provides a versatile platform for precise antibacterial therapy. MNPs exhibit unique magnetic responsiveness, enabling targeted drug delivery, magnetothermal and photothermal effects, and multimodal imaging (Allafchian and Hosseini, 2019; de Toledo et al., 2018). Under external magnetic fields, MNPs can concentrate therapeutic agents at infection sites, enhancing local efficacy while minimizing systemic side effects (Wang et al., 2018). Furthermore, their magnetothermal effect can disrupt biofilms and trigger on-demand drug release (Marchianò et al., 2021; Shivanna et al., 2022). Through surface functionalization, MNPs can be loaded with antibiotics, photosensitizers, or metal ions, allowing microenvironment-responsive release (Wu et al., 2021; Zhong et al., 2022). Notably, their inherent MRI contrast capability supports theranostic applications (Jia et al., 2019; Zou et al., 2021).

Recent advances include bio-based core–shell structures, metal-decorated composites, and magnetically driven microrobots, which enhance antibacterial performance and enable mechanical biofilm disruption. However, clinical translation requires careful adaptation to infection site anatomy and precise control of MNP physicochemical properties.

This review highlights the principle that “infection site dictates treatment strategy,” discussing the design, energy delivery, and synergistic mechanisms of MNPs for different clinical scenarios. It also examines current challenges and future trends, aiming to guide the development of effective and safe MNP-based antibacterial systems.

This narrative review adopted a targeted and rigorous literature selection strategy to ensure the relevance, authority and reliability of the included studies. Relevant peer-reviewed articles, experimental investigations and clinical reports published within the recent 10 years were retrieved from mainstream academic databases, including PubMed, Web of Science, Scopus and Google Scholar, using core keywords such as magnetic nanomaterials, antibacterial therapy, infection-site targeted treatment, bone infection repair, wound healing (customizable core keywords). Duplicate records, low-quality non-peer-reviewed gray literature, irrelevant studies and those with inconsistent research objectives were manually excluded. We only included high-quality studies with complete experimental datasets, standardized methodological designs and consistent research focuses, thereby ensuring a comprehensive, objective and credible overview of this research field.

## 2 Types and physicochemical properties of magnetic nanomaterials

### 2.1 Types and characteristics

Currently, iron oxide nanoparticles (IONPs) are the core materials used in antibacterial research, primarily including magnetite (Fe_3_O_4_) and maghemite (γ-Fe_2_O_3_) (Zhang and Miao, 2024). They possess superparamagnetism, good biocompatibility—with Fe_3_O_4_ being FDA-approved as a GRAS (Generally Recognized As Safe) material—and tunable surface active sites, all of which form the basis for constructing multifunctional antibacterial platforms (Al-Shabib et al., 2018). IONP size is typically controlled between 6 and 120 nm. Specifically, smaller particles (6–9 nm) exhibit more significant direct antibacterial activity due to their larger specific surface area and stronger iron ion release capability; in contrast, larger particles (50–120 nm) possess higher magnetothermal conversion efficiency, making them more suitable for magnetothermal therapy (Kudr et al., 2017). Smaller particles provide higher surface-to-volume ratios for ligand attachment, whereas multicore or anisotropic structures can enhance magnetic responsiveness and catalytic activity. It may be remarkable to discuss alternative nanoparticle morphologies as cubic (Wang Q. et al., 2020), rod-shaped (Marcuello et al., 2018) or nanoflowers (Theodosiou et al., 2022) and how the size and shape of the magnetic nanoparticles directly rely on the magnetic saturation and the effective surface area for further bioconjugation procedures. Beyond conventional spherical nanoparticles, alternative morphologies including cubic, rod-shaped, and nanoflower-like MNPs have demonstrated distinct magnetic and biological properties. Cubic nanoparticles exhibit higher magnetic anisotropy and enhanced magnetic saturation due to shape-dependent crystal facet exposure, improving magnetothermal efficiency (Egea-Benavente et al., 2023). Rod-shaped nanoparticles provide increased aspect ratios that enhance magnetic responsiveness and facilitate mechanical interaction with bacterial membranes (Zhang and Miao, 2024). Nanoflower structures, composed of clustered nanocrystals, offer exceptionally high surface area and magnetic susceptibility, enabling improved drug loading capacity and magnetic hyperthermia performance (Shen et al., 2024). Importantly, nanoparticle size and morphology directly influence saturation magnetization, heat generation efficiency, and effective surface area available for bioconjugation.

Composite MNP systems based on IONPs mainly include three categories: 1) Metal-decorated types (e.g., Ag-, Cu-, or Au-decorated IONPs), synergizing the oxidative stress effect of IONPs with the enzyme inhibition and DNA disruption effects of metal ions (Tasnim et al., 2024); 2) Polymer composite types (e.g., composites with chitosan, PLGA, or hydrogels), improving colloidal stability, drug loading capacity, and *in vivo* circulation time (Leena Panigrahi et al., 2023); 3) Bio-functionalized types enhancing antibacterial penetration by enzymatically degrading the biofilm matrix (Song et al., 2019). Moreover, magnetic assemblies and nanorobots (e.g., spherical multi-core assemblies, helical microrobots, anisotropic multicore IONP assemblies) with controllable motility enable mechanical biofilm disruption and precise drug delivery, emerging as novel antibacterial carriers (Gao et al., 2025). Notably, anisotropic multicore IONP assemblies (microrobots) composed of multiple small iron oxide nanoparticles aligned under an external magnetic field and fixed with an inorganic or polymeric matrix have attracted increasing attention, as they can perform complex motion in response to rotating magnetic fields due to their high magnetic susceptibility (Tran et al., 2025).

### 2.2 Strategies for regulating physicochemical properties

Size and morphology control are core means to optimize MNP antibacterial efficacy. Spherical IONPs facilitate uniform dispersion and *in vivo* clearance, whereas anisotropic structures like rods and plates can enhance photothermal conversion efficiency and interaction with bacterial surfaces. For instance, copper ferrite (CuFe_2_O_4_) nanosheets exhibit a 30% increase in Fenton-like reaction activity and a photothermal conversion efficiency (η) of 42.6% compared to spherical particles, significantly enhancing synergistic antibacterial effects (Liu et al., 2019) (Table 1). Uniformity control can reduce batch variations; optimizing reaction parameters via solvothermal or co-precipitation methods can control the IONP size distribution coefficient within 0.15, ensuring reproducible antibacterial effects (Zhang and Miao, 2024).

**TABLE 1:** Magnetic metal-based nanomaterials: antibacterial activity, mechanisms and application parameters (Zhong et al., 2022).

Category	Metal-based nanomaterial	Activity	Main antibacterial mechanism	Infection site	Pathogen or biofilm model	Magnetic modality and key parameters	Route of administration	References
Oxide	ε-Fe_2_O_3_ NPs	Magnetothermal Activity	Magnetothermal therapy; magnetic targeting	Cutaneous soft tissue infection, peri-implant osteomyelitis	*Staphylococcus aureus*	AMF-induced magnetothermal therapy + static magnetic targeting; Frequency: 20–100 kHz, Field amplitude: 4–95 kA/m; local hyperthermia for biofilm/bacterial eradication	Local injection (infection focus/femoral canal); topical cutaneous delivery	Fang et al. (2017), Gu et al. (2020), Kim et al. (2013), Yuwen et al. (2025)
​	Fe_3_O_4_ NPs	Magnetothermal Activity and Magnetophysical Activity	Magnetothermal therapy, magnetic targeting therapy	Cutaneous wound infection, superficial soft tissue infection, peri-implant osteomyelitis	*Staphylococcus aureus* (MRSA)	AMF hyperthermia + static magnetic targeting (ultrasound release); 150–300 kHz, 10–100 kA/m, H⋅f ≤ 5 × 109 A/(m⋅s) 42 °C–47 °C, 15–30 min	Local infection injection, topical wound/implant application, IV injection with external magnetic targeting	Fang et al. (2017), Li et al. (2020), Zhang et al. (2019)
​	CuFe_2_O_4_ Nanosheets	Photothermal Activity and Peroxidase-Like Activity	Enhanced photothermal conversion efficiency	Cutaneous wound infection, infectious bone defect, superficial soft tissue infection	*Staphylococcus aureus*, *Pseudomonas aeruginosa*	PTT + peroxidase/CDT; NIR laser (808 nm/1064 nm); local temperature 42 °C–48 °C, 10–20 min	Topical wound/defect application, local infection injection, hydrogel-based localized implantation	Liu et al. (2019), Zhang X. et al. (2024)
Composite	Mg_2_SiO_4_-CuFe_2_O_4_ Core-Shell NPs	Magnetothermal Activity	Magnetothermal therapy; enhanced stability and targeting	Infectious bone defect, peri-implant osteomyelitis, cutaneous wound infection, superficial soft tissue infection	*Staphylococcus aureus*, *Pseudomonas aeruginosa*	AMF magnetic hyperthermia; 150–300 kHz, 10–100 kA/m; biosafety threshold H⋅f ≤ 5 × 109 A/(m⋅s); 42 °C–47 °C, 15–30 min; static magnetic targeting; high-efficiency spinel ferrite NPs	Composite scaffold implantation, local infection injection, hydrogel-based topical delivery, topical wound application	Bigham et al. (2019); Bigham et al. (2021); Abdelrahman et al. (2024); Fotukian et al. (2020)
​	Fe_3_O_4_@Bi_2_S_3_ Urchin-like Spheres	Magnetothermal Activity and Magnetophysical Activity	Physical disruption; magnetothermal therapy; immune stimulation	Implant infection	*In vitro* bacterial biofilm model	RMF physical disruption + magnetothermal therapy; propeller-like rotation for biofilm destruction	Local injection	Xu et al. (2024)
​	NiFe_2_O_4_@Au Core-Shell NPs	Photothermal Activity	Photothermal therapy; enhanced thermal conversion efficiency	No *in vivo* infection route tested	*Staphylococcus aureus*, *Escherichia coli*	External static magnetic field targeting; magneto-mechanical photothermal synergy; magnetic field disrupts bacterial membranes to boost hyperthermia	No *in vivo* administration route tested	Xu et al. (2022)
​	γ-Fe_2_O_3_/Ag Hybrid NPs	Magnetothermal Activity	Magnetothermal therapy; Ag-enhanced antibacterial activity	no *in vivo* infection site tested	*Klebsiella pneumoniae*	AMF-induced magnetic hyperthermia; superparamagnetic γ-Fe_2_O_3_ core; tunable AMF parameters for high heating efficiency; magnetic targeting potential	no *in vivo* administration route tested	Lemine et al. (2021); Luengo et al. (2020)
​	CoFe_2_O_4_/MnFe_2_O_4_	Magnetothermal Activity	Magnetothermal therapy	soft-tissue infectious sites	Planktonic Gram+/Gram− bacteria	AMF-induced magnetothermal effect; hard-soft bi-magnetic core-shell; exchange anisotropy tuning; tunable AMF	local injection; magnet-guided intravenous injection	Ansari et al. (2023); Fiaz et al. (2023); Narayanaswamy et al. (2022); Nica et al. (2020)
​	Fe_3_O_4_/Carbon Nanotube Composite	Magnetothermal Activity	Microwave thermal therapy; magnetic targeting therapy	MRSA-infected osteomyelitis; deep tissue lesions	MRSA; planktonic bacteria	External static magnetic targeting; microwave-induced magnetothermal effect; focal infection enrichment	Local injection; magnet-guided intravenous injection	Qiao et al. (2020); Sabik et al. (2024)
​	Fe_3_O_4_/ZIF-8 Coating	Magnetothermal Activity and Ion Release Activity	pH-responsive Zn^2+^ release for sterilization; magnetothermal therapy	Infected skin wounds; jaw osteomyelitis	*S. Aureus*;planktonic bacteria	Static magnetic targeting; pH-triggered ZIF-8 degradation (Zn^2+^ release); magnetothermal therapy; superparamagnetic Fe_3_O_4_-dominated core-shell nanostructures	Topical wound coating; local lesion administration	Chen et al., 2018; Li H. et al. (2026); Zhang J. et al. (2025)
​	pFe_3_O_4_@GOx	Peroxidase-Like Activity	Generates H_2_O_2_ for chemodynamic therapy; lowers pH; magnetic retention	Bacteria-infected skin wounds	*Staphylococcus aureus*, *Escherichia coli*	Superparamagnetic pFe_3_O_4_; external static magnetic field for magnetic retention	Topical wound application	Gong et al. (2021)

Surface modification and functionalization enable optimization of MNP targeting, responsiveness, and biocompatibility (Araújo et al., 2024). Intravenous delivery systems employ PEGylation, cell membrane coating, etc., to prolong *in vivo* circulation time to over 24 h (Halevas and Varna, 2025). In contrast, local application systems modulate surface charge via amino or carboxyl group modification; positively charged MNPs can adsorb onto negatively charged bacterial surfaces via electrostatic interactions, enhancing local enrichment (Kralj et al., 2011). Responsive modifications (e.g., pH-sensitive ZIF-8, enzyme-sensitive hyaluronic acid) enable on-demand release of antibacterial components, avoiding systemic toxicity (Obaid et al., 2024). Moreover, surface hydrophobicity modulation, such as oleic acid and rhamnolipid functionalization, can effectively inhibit bacterial adhesion and disrupt preformed biofilms (Nickzad and Déziel, 2014; Stenz et al., 2008).

Magnetic property regulation focuses on improving magnetothermal conversion efficiency and magnetically guided targeting precision. Doping with elements like Co or Mn to prepare composite ferrites (e.g., CoFe_2_O_4_, MnFe_2_O_4_) can increase specific loss power (SLP) to over 80 W·g^-1^, significantly reducing the required MNP dose for magnetothermal therapy (Fiaz et al., 2023) (Table 1). Magnetic field-assisted assembly into magnetic nanochains can enhance magnetic response force, enabling rapid enrichment at infection sites under a static magnetic field, with enrichment efficiency increasing 2–3 times compared to single particles, while key parameters refer to superparamagnetic Fe_3_O_4_-dominated core-shell nanostructures with no involvement of alternating magnetic field or magnetothermal therapy (Li G. et al., 2026). For anisotropic MNPs, their magnetic properties can be fully exploited by permanent magnetic actuation systems (PMAS) and electromagnetic actuation systems (EMAS), both of which are capable of generating rotating magnetic fields to realize advanced functionalities such as directional propulsion and drilling (Serantes et al., 2018).

Surface modification efficiency is a critical parameter influencing reproducibility and therapeutic performance. Reported ligand conjugation yields for PEGylation, antibiotic loading, or biomolecule coupling, depending on surface chemistry and reaction conditions (Vasić et al., 2024). Carbodiimide coupling and silane chemistry often achieve high grafting densities, whereas polymer encapsulation strategies may improve drug loading efficiency (Mudhakir et al., 2024). Quantitative reporting of modification yields is essential to ensure batch consistency and predictable biological responses.

Aggregation remains a critical issue during nanoparticle synthesis and bioconjugation. Magnetic dipole–dipole interactions and insufficient surface stabilization may lead to clustering (Karimova et al., 2023), reducing effective surface area, impairing targeting performance, and altering magnetothermal behavior. Strategies such as polymer coating, surfactant stabilization, surface charge optimization, and steric hindrance modification have been widely adopted to improve colloidal stability (Bilal et al., 2022; Schubert and Chanana, 2018). Dynamic light scattering and zeta potential measurements are commonly used to monitor aggregation behavior and dispersion stability (Rodriguez-Loya et al., 2023).

## 3 Strategies for superficial skin and tissue infections: precise external energy delivery

**FIGURE 1:**
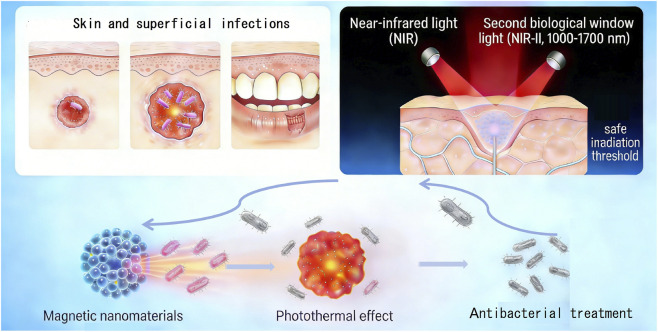
Schematic illustration of the therapeutic strategy using magnetic nanomaterials for skin and superficial infections under near-infrared light irradiation. The illustration depicts the application of magnetic nanomaterials in treating skin and superficial infections—such as burns and periodontitis—through photothermal conversion. Upon exposure to second biological window near-infrared light, the nanomaterials generate a photothermal effect, providing localized thermal therapy while operating within a safe irradiation threshold.

Infections of the skin and superficial tissues (e.g., chronic wounds, burns, periodontitis), where lesions are directly exposed or located within a few centimeters of the body surface, facilitate non-invasive external energy delivery (Figure 1). Near-infrared (NIR) light, especially in the second biological window (NIR-II, 1000–1700 nm), is the preferred external trigger for activating MNPs due to its deep tissue penetration and high safe irradiation threshold (Chen X. et al., 2025). This strategy, centered on spatiotemporal controllability, multi-mechanism synergistic antibacterial action, and potential for tissue repair promotion, combined with novel composite MNP designs, significantly improves treatment efficacy for superficial infections.

### 3.1 Clinical challenges and design requirements

Chronic wound infections (e.g., diabetic foot ulcers), burn infections, and periodontitis are often characterized by dense biofilms formed by multiple microorganisms, rendering traditional antibacterial drugs ineffective due to poor penetration and leading to recurrent episodes (Mallick et al., 2022). The design requirements for thes infection include: ① energy delivery must achieve superficial tissue penetration with high spatial confinement to avoid damage to adjacent healthy tissue; ② the delivery system must accommodate an open wound environment, providing wet adhesiveness and management of exudate; ③ material design should integrate bactericidal efficiency with tissue-repair functions to break the persistent inflammation–infection cycle in chronic wounds.

### 3.2 NIR as an ideal external excitation source

NIR light can non-invasively penetrate superficial tissue (2–3 cm), activating MNPs to generate photothermal effects (PTT), photodynamic effects (PDT), or synergistic chemodynamic effects (CDT), achieving multi-mechanism bactericidal action (Yan et al., 2023). NIR-II window light offers lower tissue scattering and absorption compared to the traditional NIR-I window (700–900 nm), further improving energy delivery efficiency and reducing damage to normal tissue (Zhu et al., 2018). For example, rhamnolipid-coated, silver and superparamagnetic iron oxide (SPION)-modified POSS nanoparticles (RSMP) achieved inhibition rates of 90% and 66% against *S. aureus* and *P. aeruginosa*, respectively, under NIR irradiation, and supported human osteoblast proliferation even at a concentration of 0.05 g/L (Kibar et al., 2025).

### 3.3 Material design and formulation innovation

To effectively treat superficial infections, MNP design must focus on three aspects: maximizing NIR absorption efficiency, enhancing biofilm penetration, and tailoring the formulation to the wound microenvironment. Compounding superparamagnetic iron oxide with noble metals (Au), metal sulfides (CuS), or narrow-bandgap semiconductor materials can significantly enhance photothermal conversion efficiency in the NIR-II window (Naskar and Kim, 2022; Yougbaré et al., 2020). For instance, copper ferrite (CuFe_2_O_4_) MNPs possess good photothermal properties and Fenton-like reactivity; the contained Cu^+^/Fe^2+^ ion pairs can catalyze ·OH generation in the infection microenvironment, achieving self-enhancing synergistic PTT and CDT bactericidal action (Liu et al., 2019). Furthermore, NiO NPs@AuNPs@Van (NAV) nanocomposites leverage vancomycin-mediated self-aggregation to generate localized photothermal effects on *MRSA* surfaces, synergizing with the magnetic enrichment effect of magnetic NiO, achieving a bactericidal rate of 99.6% and accelerating infected wound healing (Du et al., 2022).

Formulation innovation ensures the local application of MNPs. Integrating functional MNPs into formulations like hydrogels, nanofiber membranes, or microneedles enables the development of smart dressings responsive to the wound microenvironment (Guo et al., 2024). Magnetic nanofiber dressings coated with polydopamine and curcumin nanocrystals first eliminate *MRSA* via photothermal action under NIR irradiation, then release curcumin to polarize macrophages toward the M2 phenotype, simultaneously achieving anti-infection and wound healing (Xu et al., 2021). Additionally, hydrogel dressings modified with highly oriented magnetic nanocomposite–protein fiber assemblies (GMPF) can generate nitric oxide (NO) catalyzed by copper ions, regulating fibroblast behavior and promoting anti-fibrotic skin regeneration (He et al., 2025).

### 3.4 From theranostics to immunomodulation

Multifunctional MNP systems are evolving toward integrated “theranostics–immunomodulation.” Fc-MBL@rGO@Fe_3_O_4_ composite MNPs combine broad-spectrum bacterial targeting recognition, peroxidase-like activity, low-power NIR-II photothermal conversion, magnetotargeting, and chromogenic response characteristics. They achieve CDT by catalyzing H_2_O_2_ into ·OH via Fe_3_O_4_, synergizing with rGO’s photothermal effect to reach a 100% bactericidal rate, while enabling visual infection monitoring within 30 min via TMB chromogenic reaction, meeting the needs for rapid emergency wound management (Xue et al., 2024). Regarding immunomodulation, magnetic nanoformulations can promote tissue repair by regulating macrophage polarization and reducing inflammatory cytokine levels. For instance, the NAV system reduced IL-6 and TNF-α levels in infected wounds while upregulating VEGF expression, accelerating skin regeneration (Du et al., 2022). Moreover, IONPs can promote the polarization of macrophages into the M1 (pro-inflammatory) subtype, enhancing their bactericidal effect on intracellular bacteria and reducing infection recurrence (Yu et al., 2020).

## 4 Strategies for deep soft tissue and bone infections: synergy of penetration and intervention

**FIGURE 2:**
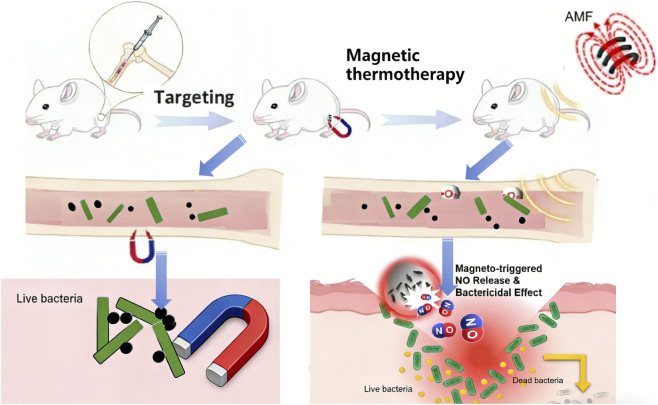
Schematic illustration of magnetically targeted and magnetothermal therapy for deep-seated infections such as osteomyelitis. This figure depicts the therapeutic mechanism of magnetic nanomaterials in treating deep-tissue bacterial infections. Magnetic targeting enables the precise accumulation of nanomaterials at the infection site (e.g., bone tissue in osteomyelitis). Upon exposure to an alternating magnetic field (AMF), the nanomaterials generate localized heat (magnetic thermotherapy). Furthermore, the magnetic stimulus can trigger the controlled release of antibacterial agents such as nitric oxide (NO). The combined magnetothermal effect and NO release lead to efficient bacterial eradication, as represented by the transition from live to dead bacteria (Ren et al., 2024).

Lesions in deep soft tissue and bone infections (e.g., osteomyelitis, abdominal abscesses, septic arthritis) are located more than 3 cm below the body surface, where NIR light energy attenuates significantly, making effective MNP activation difficult. The core treatment strategy for such infections is “penetrating physical field + intervention technology,” utilizing the deep penetration capability of AMF (no tissue attenuation) to achieve magnetothermal therapy or magnetically targeted enrichment (Figure 2), combined with image-guided intervention techniques to enhance local drug delivery and energy delivery, overcoming depth limitations (Zhang Y. et al., 2025).

### 4.1 Core challenges and design requirements

The anatomical location of deep infections poses difficulties for external energy delivery. Furthermore, biofilm formation, poor blood supply, and the difficulty of drug penetration into bone tissue/abscess cores exacerbate treatment challenges (Liu et al., 2020). For example, osteomyelitis involves dense cortical bone encapsulation that causes extremely poor permeability and drug penetration, with hypovascularity limiting passive accumulation and dense biofilms on necrotic bone forming impenetrable barriers, accompanied by translational hurdles including implant biocompatibility, sustained focal retention, and avoidance of antibiotic resistance and systemic toxicity; soft tissue abscesses feature fibrotic pseudocapsules that induce heterogeneous permeability and block drug-pathogen contact, while hypoxic-acidic niches promote dense biofilm maturation to reduce nanomaterial diffusion, with translational priorities covering minimally invasive delivery, confined retention, and prevention of systemic carrier leakage; septic arthritis entails joint cavity confinement and synovial fluid turnover that restrict intra-articular carrier retention, alongside avascular cartilage that impairs permeability and biofilm colonization on cartilage surfaces, posing translational challenges such as precise intra-articular delivery, prolonged retention, and healthy cartilage protection.The design requirements for this site are: ① energy delivery must rely on physical fields with no tissue attenuation (e.g., alternating magnetic fields, AMF) or interventional energy delivery; ② the delivery system must overcome bone tissue barriers, poor blood perfusion in abscess cores, and the multi-layer defense of biofilms; ③ material design should prioritize high specific loss power (SLP) for magnetothermal conversion and compatibility with imaging modalities to enable image-guided precision.

### 4.2 Core treatment strategy: integration of magnetic fields and intervention techniques

For deep infections, MNP antibacterial strategies shift from sole reliance on NIR light to a dual-path approach of “AMF-driven + intervention-enhanced,” leveraging both the deep penetration of AMF and the precise delivery of therapeutic agents via intervention technology, significantly improving local efficacy (Yang et al., 2024). MNP therapy can be combined with established clinical treatments to enhance therapeutic outcomes. For example, magnetothermal therapy may be integrated with surgical debridement to eliminate residual biofilms, while magnetically guided drug delivery can complement systemic antibiotic therapy by increasing local drug concentration (Nowak-Jary and Machnicka, 2022). In osteomyelitis management, combining MNP-mediated hyperthermia with antibiotic-loaded bone cement or scaffolds has demonstrated synergistic antibacterial and osteogenic effects. Additionally, integration with negative-pressure wound therapy, ultrasound-assisted drainage, or localized irrigation systems may further improve infection control.

#### 4.2.1 AMF-mediated deep magnetothermal therapy and magnetotargeting

AMF can penetrate biological tissue without attenuation, activating MNPs to generate magnetothermal effects or guiding MNP enrichment at the infection site. Magnetothermal therapy requires designing MNPs with high specific loss power (SLP), optimizing their size, morphology, and composition to improve magnetothermal conversion efficiency (Allafchian and Hosseini, 2019). For instance, Fe_3_O_4_/carbon nanotube composites generate microwave hyperthermia effects under AMF, successfully treating *MRSA*-infected osteomyelitis when combined with magnetotargeting (Qiao et al., 2020) (Table 1). Mg nanomaterial implants generate eddy current thermal effects under a low-intensity alternating magnetic field with a typical intensity of 5–20 mT, frequency of 50–200 kHz and daily exposure duration of 15–30 min, controllably releasing H_2_, OH^−^, and Mg^2+^. H_2_ scavenges ROS for anti-inflammatory effects, OH^−^ hinders bacterial energy metabolism, and Mg^2+^ promotes bone formation, achieving integrated “antibacterial–anti-inflammatory–bone repair” therapy (Yang et al., 2024).

Magnetically guided targeting technology can actively enrich intravenously or locally injected MNPs at the infection site under a static magnetic field, increasing local drug concentration. Studies confirm that a static magnetic field with moderate intensity of 0.2–0.6 T and consistent long-term exposure can significantly increase MNP coverage around osteoblasts, enhancing the accumulation of antibacterial materials at bone infection sites (Chen S. et al., 2025). FeCo alloy MNPs under a magnetic field achieved inhibition rates as high as 63% against *S. aureus* and *E. coli*, significantly higher than without a magnetic field (Moditma et al., 2021). Moreover, the “bacterially endogenous MNPs” strategy offers an innovative approach for deep infection treatment. Pathogens can biosynthesize MNPs using host iron and zinc ions; under AMF, heat generation of 5 °C–6 °C significantly reduces bacterial viability without requiring exogenous MNP delivery, avoiding carrier-related toxicity (Kaushik et al., 2022).

#### 4.2.2 Interventional energy and drug delivery

For abscess-type infections, after ultrasound/CT-guided puncture and drainage, drug-loaded nanomaterial formulations can be injected through the channel, combined with percutaneous interstitial phototherapy (fiber optic insertion into the cavity for irradiation), achieving “debridement + local intensified therapy” to reduce recurrence rates (Lv et al., 2025). Interventional techniques enable minimally invasive direct delivery of MNPs or energy delivery devices to the lesion, solving drug delivery and energy activation challenges in deep infections. In orthopedic surgery, after open debridement, magnetic nanocomposite scaffolds can be implanted into bone defect areas, followed by non-invasive activation of magnetothermal therapy via AMF postoperatively to eliminate residual microscopic lesions (Tavares et al., 2023; Wang L. et al., 2025). For example, nanoscale porous magnetic hydroxyapatite scaffolds loaded with silver nanoparticle (Ag NP)-decorated MNPs can effectively kill *MRSA* and inhibit biofilm formation while promoting bone tissue repair (Sadeghi et al., 2024). ZnFe_2_O_4_ (ZFO) nanoparticle-modified PLGA/PCL magnetic scaffolds, under a 30 mT static magnetic field, can enhance the expression of osteogenesis-related genes while exerting antibacterial effects via Zn^2+^ and Fe^3+^ and clearing ROS to improve the repair microenvironment (Chen S. et al., 2025).

### 4.3 Multimodal imaging-guided precision theranostics

The compatibility of MRI with MNPs supports precision theranostics for deep infections. MRI can non-invasively determine infection extent (e.g., bone marrow edema, abscess wall), assess MNP targeting and enrichment, and monitor lesion changes during treatment in real-time (Dong et al., 2017). For instance, a dual-functional probe constructed from Gd-BBDC1.25 metal–organic frameworks (MOFs) modified with Ag NPs enables MRI imaging of bacteria as low as 10^5^ CFU and simultaneous *in situ* antibacterial therapy (Yu et al., 2025). SPIONs can serve as contrast agents for both MRI and magnetic particle imaging (MPI), guiding magnetothermal therapy implementation while regulating bacterial transcription via temperature-sensitive repressor proteins, offering a new pathway for targeted therapy (Greeson et al., 2022). Additionally, magnetic particle imaging (MPI) with high sensitivity and specificity can provide real-time tracking of MNP distribution in deep tissues, further improving the precision of theranostics (Greeson et al., 2022).

## 5 Strategies for cavity organ and implant-related infections: precision interface intervention

**FIGURE 3:**
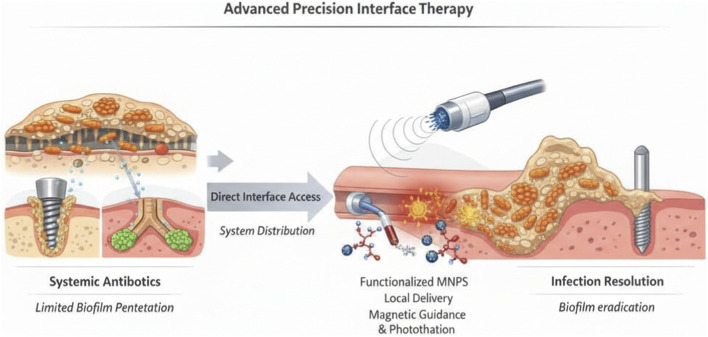
Precision interface therapy for biofilm-associated infections. Biofilm formation on implant surfaces and luminal interfaces creates an extracellular polymeric substance (EPS) barrier that impedes antibiotic penetration and protects bacteria from immune clearance, leading to persistent infection and recurrence. Magnetic nanoparticle (MNP)–enabled precision interface therapy integrates surface defense, magnetic activation, and targeted local delivery to overcome these barriers. Functionalized interfaces inhibit bacterial adhesion and disrupt early biofilm formation, while magnetically responsive nanoparticles enable localized magnetothermal effects, reactive oxygen species (ROS) generation, and mechanical disruption of the EPS matrix to enhance antimicrobial penetration. Concurrently, site-specific delivery concentrates therapeutics at the infection interface while minimizing systemic exposure. These coordinated strategies eradicate biofilms, control infection, and promote tissue healing and implant integration.

Infections related to cavity organs and implants (e.g., ventilator-associated pneumonia, catheter infections, orthopedic implant infections) involve lesions located on the inner wall of lumens or at the implant–tissue interface. Traditional systemic drug administration struggles to penetrate biofilms to reach the infection site, often leading to treatment failure and recurrence (Steckiewicz and Inkielewicz-Stepniak, 2020; Tran and Tran, 2012). The treatment strategy for such infections needs to shift from “systemic distribution” to “direct interface access,” achieving precise biofilm clearance and infection prevention through MNP surface functionalization, local intracavitary drug delivery, and remote energy activation (Figure 3).

### 5.1 Core challenges and design requirements

Infections related to cavity organs and implants (e.g., ventilator-associated pneumonia, catheter infections, orthopedic implant infections) involve lesions located on the inner wall of lumens or at the implant–tissue interface (Dong et al., 2021). The core design requirements for this site are: ① systemic drug delivery struggles to penetrate biofilms to reach the infection site, necessitating a shift from “systemic distribution” to “direct interface access”; ② material design must integrate both “anti-adhesion” and “responsive eradication” functions to counter the extracellular polymeric substance (EPS) barrier of biofilms; ③ actuation modes must adapt to local anatomy, supporting endoscopic delivery or remote magnetic field actuation.

### 5.2 Core treatment strategy: interface functionalization and precise delivery

The therapeutic core for interface infections involves constructing an integrated “active defense–responsive therapy” system, achieving precise intervention at the infection interface through MNP surface functionalization of devices, local intracavitary delivery of MNP formulations, and remote energy activation (Tran and Tran, 2012).

#### 5.2.1 Device surface functionalization: constructing smart defense interfaces

Constructing MNP composite coatings on medical device surfaces can impart antibacterial, anti-adhesion, and responsive therapeutic capabilities. Basic antibacterial coatings inhibit bacterial adhesion via contact killing or photocatalytic ROS generation, such as Ag NP- or TiO_2_-modified magnetic composite coatings effectively preventing early biofilm formation (Aswini et al., 2024; Besinis et al., 2017). More intelligent responsive coatings can sense the infection microenvironment and trigger therapy. For example, magnetically controlled pH-responsive Fe_3_O_4_/ZIF-8 coatings remain stable at physiological pH. When bacterial metabolism lowers the local pH, ZIF-8 degrades to release Zn^2+^ for bactericidal action while exposing Fe_3_O_4_ MNPs. Under AMF, these MNPs generate heat to clear mature biofilms and physically disrupt the EPS structure, thereby enhancing antibiotic penetration (Zhang J. et al., 2025) (Table 1). Silk fibroin (SFMA)-modified Fe_3_O_4_ nanoparticle coatings stably adhere to titanium surfaces in dry environments, while adhesion decreases in aqueous environments, allowing biofilm removal via external magnetic field with an intensity of 0.3–0.8 T, zero frequency and short exposure duration for field-assisted coating stripping, enhancing antibiotic efficacy 100-fold and resolving the contradiction between coating adhesion stability and magnetic responsiveness (Quan et al., 2024). Clustered magnetic microrobots composed of ferromagnetic material (Fe_3_O_4_) and photoactive material (BiVO_4_) modified on titanium dental implant surfaces can move under a transverse rotating magnetic field drive while generating ROS via BiVO_4_ to clear biofilms, providing a novel solution for dental implant infection treatment (Mayorga-Martinez et al., 2022). Moreover, anisotropic multicore IONP assemblies (microrobots) modified on implant surfaces can perform torque-driven multidirectional movement under rotating magnetic fields, enabling mechanical disruption of biofilms (Quan et al., 2024).

#### 5.2.2 Local intracavitary drug delivery and energy delivery

For established intracavitary infections, MNP formulations can be directly delivered to the infected mucosal surface via endoscopes (bronchoscope, cystoscope), combined with remote energy activation for precise therapy (Dobretsov et al., 2015). For example, researchers adsorbed vancomycin onto magnetic nanoparticle cores and spray-dried them into lactose/dextran carriers to prepare inhalable dry powder formulations for local pulmonary infection delivery, targeting drug deposition in lung tissue and reducing systemic side effects (Huang et al., 2025). Magnetic field-based energy delivery technology can activate intracavitary MNPs for physical bactericidal action, avoiding repeated interventional procedures. For deep-seated implants, MNP coatings or formulations around the implant can be activated via external AMF applied to the body surface, inducing magnetothermal therapy or controlled drug release (Anderson et al., 2019; Coffel and Nuxoll, 2015; Tran and Tran, 2012). For instance, drug-loaded magnetic nanoparticles under AMF release vancomycin on-demand (∼8.4 ± 1.1% every 10 min), inhibiting bacterial growth for up to 4 days (Huang et al., 2025). Fe_3_O_4_@Bi_2_S_3_ sea urchin-like core–shell nanospheres under a rotating magnetic field (RMF) spiral and rotate, physically disrupting biofilms while promoting ROS generation via magnetothermal effects, stimulating macrophage polarization toward the M1 phenotype to clear dormant bacteria and inhibit infection recurrence (Xu et al., 2024) (Table 1). Additionally, magnetic microrobot swarms can be guided by external magnetic fields to penetrate deep into cavity organs, achieving precise drug delivery and mechanical biofilm disruption (Unni et al., 2020).

### 5.3 Typical application paradigms

Integrated “anti-fouling–therapy” implants represent an important development direction in this field. Their surface coatings passively resist bacterial adhesion via nanostructures or antibacterial components while incorporating MNPs for responsive therapy. Interventional precision debridement targets *P. aeruginosa* biofilms in the bronchi of cystic fibrosis patients by bronchoscopic spraying of chitosan-modified Fe-ICGCDs photosensitive magnetic composite material, followed by endoscopic fiber optic irradiation, achieving “precision debridement” of biofilms (Gao et al., 2024). Furthermore, magnetically controlled multifunctional micromotors, using H_2_O_2_ as fuel and MnO_2_ as a catalyst, drill into biofilm EPS with bubble assistance, disrupting structure while generating ·OH to kill bacteria, capable of clearing microbial fouling in microchannels within 10 min (Ji et al., 2022). Magnetic urchin-like capsule robot (MUCR) swarms have also been successfully applied for biofilm eradication on biliary stents, achieving efficient biofilm removal within a short time (Sun et al., 2022).

## 6 Synergistic antibacterial mechanisms and novel systems of magnetic nanomaterials

The antibacterial efficacy of MNPs can be further enhanced through multi-mechanism synergy. Combining functional units such as metal ions, enzymes, antibiotics, and immunomodulators to construct composite systems overcomes the limitations of single mechanisms and reduces the risk of resistance development. Novel MNP system designs are evolving toward “multifunctional integration, microenvironment responsiveness, and antibiotic-free dependence.”

### 6.1 Core synergistic antibacterial mechanisms

Synergistic antibacterial mechanisms of MNPs primarily involve the combination of magnetothermal/magnetophysical and chemical antibacterial actions with immunomodulation, enhancing bactericidal efficiency and delaying resistance development through multiple action targets. Among these, magnetothermal and magnetophysical impacts are exclusive to magnetic nanoplatforms, whereas chemical antibacterial activity and immunomodulation belong to universal nanomaterial traits, together boosting comprehensive bactericidal performance. Magnetothermal therapy combined with antibiotics can enhance drug penetration by disrupting biofilm structure via thermal effects. For example, IONPs + AMF combined with ciprofloxacin improved bactericidal efficacy against *S. aureus* biofilms by over 10-fold compared to antibiotic alone (Barzegar et al., 2025). CDT combined with MNPs forms self-powered systems. For example, pFe_3_O_4_@GOx nanogenerators convert glucose to H_2_O_2_ and gluconic acid via glucose oxidase (GOx); H_2_O_2_ is supplied to Fe_3_O_4_ to catalyze ·OH generation for CDT, while gluconic acid lowers pH to enhance catalytic activity, and magnetic retention enables localized long-term therapy (Gong et al., 2021).

Immunomodulatory synergistic mechanisms can activate host immune functions to clear bacteria. For instance, IONPs promote macrophage polarization toward the M1 phenotype, enhancing phagocytic capacity for intracellular bacteria and reducing infection recurrence (Yu et al., 2020). Virus-like magnetic mesoporous silica nanoparticles (MagParV) serve as vaccine delivery platforms, efficiently inducing specific antibody production under AMF to activate the immune system against fungal, bacterial, and viral infections (Liu et al., 2023). Additionally, magnetic nanomaterials can synergize with photodynamic therapy and chemotherapy, such as Au@Bi_2_S_3_ core–shell nanocomposites generating a large amount of ROS under 808 nm laser stimulation while exhibiting excellent photothermal conversion performance, achieving synergistic photothermal and photodynamic antibacterial effects (Wang W. et al., 2020).

### 6.2 Novel magnetic nanomaterial systems

Bio-based MNP systems have garnered wide attention due to their good biocompatibility and degradability. For example, cellulose gum (carboxymethyl cellulose, CMC) magnetic composites (Fe_3_O_4_@CMC/AgNPs) stabilize Ag NPs greenly via Euphorbia plant extracts, exhibiting excellent bactericidal effects against urinary tract infection pathogens and good biocompatibility (Pourrafsanjani et al., 2024). Moringa oleifera gum-assisted synthesized CoAg_x_Fe_2-x_O_4_ nanohybrids show significantly enhanced antibacterial, anti-biofilm, and antioxidant activities with increasing Ag content, offering new options for biomedical applications (Tamboli et al., 2024).

Stimuli-responsive MNP systems enable precise on-demand therapy. For instance, the HA-CP@Fe_3_O_4_ nano antibacterial system stabilizes copper peroxide (CuO_2_) via hyaluronic acid (HA) coating. Under bacterial-secreted hyaluronidase, HA degrades, releasing CuO_2_@Fe_3_O_4_, which self-supplies H_2_O_2_ and generates ROS via Fenton/Russell reactions, achieving efficient antibacterial action and accelerated wound healing even at low concentrations (30 μg/mL) (Zhang S. et al., 2024). Magnetic microswarm systems (p-Fe_3_O_4_ swarm) driven by rotating magnetic fields combine chemical catalysis (·OH production) and physical disruption (collective motion) to synergistically clear biofilms, capable of removing biofilm blockages from 2D surfaces and 3D U-shaped tubes along geometric paths (Gong et al., 2021).

Antibiotic-free MNP systems provide new pathways to address resistance. For example, AgCuO_2_ nanoparticles prepared via simple, low-cost techniques exhibit excellent antibacterial activity against both Gram-positive (*Bacillus subtilis*, *S. aureus*) and Gram-negative (*E. coli*, *P. aeruginosa*) bacteria, with inhibition zone diameters of 10–14 mm, and can be easily separated and recovered via magnetic fields (Ateia et al., 2023). Hesperidin conjugated with cinnamic acid-based magnetic nanoparticles shows significant bactericidal activity against multidrug-resistant Gram-positive and Gram-negative bacteria, whereas hesperidin alone exhibits no antibacterial effect (Akbar et al., 2022). Moreover, catalytic antimicrobial robots (CARs) assembled from IONPs can achieve efficient biofilm removal through mechanical brushing and catalytic killing, providing a new direction for antibiotic-free therapy (Hwang et al., 2019).

## 7 Preclinical and clinical research progress

### 7.1 Key breakthroughs in preclinical research

Currently, MNP antibacterial systems have shown significant progress in chronic wounds, osteomyelitis, and implant-related infections in animal models. In *MRSA*-infected mouse wounds, NAV nanocomposites demonstrated significantly superior therapeutic effects compared to the vancomycin group, shortening wound healing time by 40% without obvious liver or kidney function damage (Du et al., 2022). Fe_3_O_4_/carbon nanotube composites combined with AMF for treating *MRSA* osteomyelitis in rats reduced bacterial load in bone tissue by 4-log_10_ and increased bone repair rate by 50% (Qiao et al., 2020). Furthermore, optimization of magnetothermal therapy parameters enables precise temperature control within 43 °C–45 °C, avoiding damage to normal tissue and laying the foundation for clinical translation (Abu-Ayyad et al., 2024; Rytov et al., 2022). In addition, magnetic microrobots have shown minimal cytotoxicity toward both cancerous and noncancerous cell lines in vitro studies, confirming their biocompatibility (Song et al., 2022).

### 7.2 Current status and bottlenecks in clinical research

Currently, most MNP antibacterial systems are in the preclinical research stage, with few entering clinical trials, mainly focusing on local delivery systems of antibiotic-loaded magnetic nanoparticles. Such a dual-responsive magnetic delivery platform exhibits markedly improved biofilm drug penetration and antibacterial efficacy over traditional therapy, although further preclinical and clinical verification is needed to confirm its translational potential.

Current core bottlenecks for clinical translation include: 1) Insufficient miniaturization and standardization of dedicated treatment equipment; high-power lasers and large-volume AMF generators are difficult to adapt to bedside or outpatient settings (Zhang and Lu, 2024); 2) Lack of personalized treatment protocols; parameters like MNP dose, magnetic field strength/frequency are highly influenced by infection type and patient constitution, necessitating standardized evaluation systems (Stigliano et al., 2016); 3) Incomplete regulatory frameworks; as novel therapeutic products, MNPs lack targeted safety and efficacy evaluation standards, and health economic assessments are insufficient (Riaz et al., 2025); 4) Biological barriers such as biofilm heterogeneity and toxicological risks of MNPs, including mechanical damage from particle aggregation and excessive release of iron ions (Luengo et al., 2022).

## 8 Design, challenges, and biosafety of magnetic nanomaterials

Despite the great potential of MNP antibacterial systems, translation from the laboratory to the clinic faces multiple bottlenecks in material design, biosafety, and clinical translation, requiring breakthroughs via precise design and systematic evaluation.

### 8.1 Core challenges in material design

Multifunctional integration design requires balancing performance and biocompatibility. Ideal antibacterial MNPs need to simultaneously possess strong magnetism, high energy conversion efficiency, catalytic activity, and good biocompatibility. However, optimizing these properties often involves trade-offs; for instance, increasing particle size to enhance magnetothermal performance may affect *in vivo* clearance, and metal doping to enhance antibacterial activity may increase toxicity (Guo et al., 2016). Surface engineering must be adapted to the administration route; intravenous delivery requires “stealth” coatings like PEGylation to reduce immune clearance and enable active targeting, while implant coatings require strong adhesion to the substrate and long-term stability (Chand and Vasquez-Guardado, 2025; Halevas and Varna, 2025). Insufficient scalability and standardization of production; synthesis of many high-performance MNPs involves complex steps and large batch variations, lacking unified quality control standards (size distribution, magnetic parameters, sterility), hindering clinical translation (Ali et al., 2021). Additionally, for anisotropic MNPs, small synthesis deviations can lead to substantial differences in actuation behavior, affecting reproducibility (Serantes et al., 2018).

### 8.2 Biosafety considerations

#### 8.2.1 Short-term toxicity

Acute cytotoxicity and organ damage pose primary safety risks. High doses or poorly degradable coatings may induce hepatotoxicity, oxidative stress, and inflammation. However, optimized formulations like RSMP have demonstrated low toxicity to human cells via MTT assays, validating the biocompatibility of rationally designed nano-systems (Kibar et al., 2025).

#### 8.2.2 Long-term biodistribution

Systemically administered MNPs are readily sequestered by the reticuloendothelial system, leading to long-term retention in the liver, spleen, and bone marrow (Nowak-Jary and Machnicka, 2022). This persistent accumulation raises concerns about chronic tissue irritation. Surface modification with biodegradable polymers or biomimetic membranes has been verified to reduce off-target retention (Nowak-Jary and Machnicka, 2023), necessitating long-term biodistribution studies for risk mitigation.

#### 8.2.3 Metabolism and clearance

Particle fate is dictated by size, composition, and coating. Small, modified MNPs are primarily cleared via the kidneys, whereas larger aggregates are trapped in the liver and spleen (Nowak-Jary and Machnicka, 2022). Degradation products of coating materials also require monitoring. Fully elucidating metabolic pathways and excretion routes is critical to prevent unintended chronic accumulation.

#### 8.2.4 Local & systemic exposure

Excessive systemic exposure amplifies organ toxicity risks. Locally, magnetothermal therapy demands precise temperature control; insufficient heat reduces efficacy, while excess heat damages normal tissues (Baldea et al., 2025). Strict control over dosing and delivery precision is required to balance targeted killing with healthy tissue preservation.

#### 8.2.5 Immune responses

MNPs can trigger complement activation and release of pro-inflammatory factors. Iron ions and coating degradation may further induce oxidative stress, exacerbating immune-related tissue damage (Sheptulina et al., 2026). Comprehensive long-term animal studies are essential to characterize these inflammatory cascades and improve safety.

#### 8.2.6 Magnetic stimulation safety

Strict control of alternating magnetic field (AMF) parameters is mandatory. Strong AMF may cause eddy current heating in non-target tissues (Pilpilidis et al., 2025). Real-time temperature monitoring systems must be developed to ensure therapeutic windows are both efficacious and safe.

### 8.3 Optimization strategies

Upgraded surface functionalization can improve biocompatibility and targeting. Using biodegradable coatings (e.g., chitosan, alginate) or biomimetic modifications (e.g., cell membrane coating) can reduce immune clearance and organ accumulation (Wang J. et al., 2025). The integration of magnetic field technology and imaging technology, developing multi-axis electromagnetic coil systems for precise regulation of magnetic field strength and frequency, combined with real-time MRI/MPI monitoring of MNP distribution and therapeutic effect. Multi-modal synergistic design integrating magnetothermal therapy, photothermal therapy, immunotherapy, etc., can enhance therapeutic efficacy against complex biofilm infections and reduce dependence on single-treatment doses (Almeida et al., 2022; Qi and Tay, 2025). Additionally, developing simplified magnetic actuation systems and standardized fabrication methods for MNPs can improve scalability and reproducibility (Sun et al., 2024).

## 9 Conclusion and future perspectives

This review systematically summarizes precision strategies using magnetic nanoparticles (MNPs) for treating bacterial infections across diverse anatomical sites. The therapeutic efficacy of MNPs depends on the infection context and is optimized through precise control of their physicochemical properties. Material systems and energy delivery modalities must be tailored to the specific anatomical and pathological characteristics of superficial, deep-tissue, and cavity/implant-interface infections. By combining magnetic targeting, magneto/photothermal effects, multifunctional drug delivery, and multimodal imaging, MNPs provide versatile solutions for drug-resistant and biofilm-associated infections, showing particular promise in chronic wounds, osteomyelitis, and implant-related infections.

Future development of MNP-based antibacterial therapy will focus on intelligent design, theranostic integration, synergistic enhancement, and multi-technology convergence. Intelligent MNPs will respond dynamically to multiple signals within the infection microenvironment, enabling cascaded responsiveness and feedback regulation. Theranostic platforms will integrate high-resolution multimodal imaging with therapy, forming a closed-loop system for image-guided intervention and real-time monitoring. Synergistic approaches may combine MNP-mediated physical antibacterial effects with immunotherapy or phage therapy, while convergence with flexible electronics, microfluidics, and magnetically controlled microrobots will drive antibacterial therapy toward digitization and personalized medicine.

Critical challenges remain, including biocompatibility optimization, precise magnetic field control, scalable production, and clinical translation. Advances in surface biomimetic modification, improved magnetic field systems, and multimodal synergistic design will enhance safety and efficacy. Establishing standardized evaluation and regulatory frameworks is essential to facilitate the transition from bench to bedside.

Future research priorities include: (i) Scalable and reproducible synthesis strategies to ensure batch-to-batch consistency. (ii)Advanced surface engineering to improve targeting specificity and immune compatibility. (iii)Comprehensive long-term studies on biosafety, biodistribution, and pharmacokinetics. (iv)Integration with multimodal therapeutic approaches for synergistic infection control. (v)Development of clinically translatable magnetic guidance and imaging systems.

Addressing these directions will accelerate the translation of MNP-based antimicrobial platforms from experimental research to clinical applications, positioning MNPs as a transformative tool against the global antibiotic resistance crisis.
